# Galactolipid and Phospholipid Profile and Proteome Alterations in Soybean Leaves at the Onset of Salt Stress

**DOI:** 10.3389/fpls.2021.644408

**Published:** 2021-03-17

**Authors:** Ailin Liu, Zhixia Xiao, Zhili Wang, Hon-Ming Lam, Mee-Len Chye

**Affiliations:** ^1^School of Biological Sciences, The University of Hong Kong, Pokfulam, China; ^2^Centre for Soybean Research of the State Key Laboratory of Agrobiotechnology and School of Life Sciences, The Chinese University of Hong Kong, Shatin, China

**Keywords:** *Glycine max*, lipid profiling, lipid signaling, membrane lipids, metabolism, salinity

## Abstract

Salinity is a major environmental factor that constrains soybean yield and grain quality. Given our past observations using the salt-sensitive soybean (*Glycine max* [L.] Merr.) accession C08 on its early responses to salinity and salt-induced transcriptomic modifications, the aim of this study was to assess the lipid profile changes in this cultivar before and after short-term salt stress, and to explore the adaptive mechanisms underpinning lipid homeostasis. To this end, lipid profiling and proteomic analyses were performed on the leaves of soybean seedlings subjected to salt treatment for 0, 0.5, 1, and 2 h. Our results revealed that short-term salt stress caused dynamic lipid alterations resulting in recycling for both galactolipids and phospholipids. A comprehensive understanding of membrane lipid adaption following salt treatment was achieved by combining time-dependent lipidomic and proteomic data. Proteins involved in phosphoinositide synthesis and turnover were upregulated at the onset of salt treatment. Salinity-induced lipid recycling was shown to enhance jasmonic acid and phosphatidylinositol biosyntheses. Our study demonstrated that salt stress resulted in a remodeling of membrane lipid composition and an alteration in membrane lipids associated with lipid signaling and metabolism in C08 leaves.

## Introduction

Soybean (*Glycine max* [L.] Merr.) is one of the most important legume crops used as food and feed (Hasanuzzaman et al., 2016). As a major environmental constraint on crop yield, salinity triggers osmotic stress in roots followed by whole-plant ionic toxicity arising from Na^+^ and Cl^–^ ion accumulation (Munns and Tester, 2008). Plants have evolved to adapt to high salt by salt-responsive signaling, balancing osmotic pressure in organic osmolyte formation, Na^+^ compartmentalization, enhanced scavenging of reactive oxygen species (ROS), phytohormone regulation, and cell structure modulation (Parvaiz and Satyawati, 2008; Phang et al., 2008). As modifying in membrane lipid composition provides an effective strategy in maintaining cell membrane integrity to confront stress (Elkahoui et al., 2004), the characterization of membrane lipids following salt treatment is crucial in formulating strategies to overcome salinity. Qualitative and quantitative lipid profiling following abiotic stresses such as drought (Gigon et al., 2004), cold (Barrero-Sicilia et al., 2017), or heat (Higashi et al., 2015) have been studied in Arabidopsis. Furthermore, salt-induced membrane lipid alterations documented in salt-sensitive varieties of *Arabidopsis thaliana* and *Triticum sativum*, and salt-tolerant varieties of *Brassica napus* and *Zea mays* indicated that adaptation to salinity stress relied on total membrane lipid composition (Guo et al., 2019). Other reports revealed a reduction in membrane lipids in the salt-sensitive barley after salinity stress (*Hordeum vulgare* L. cv. Manel) while no changes were evident in the salt-tolerant *Hordeum maritimum* and the halophyte *Suaeda altissima* (Chalbi et al., 2013; Tsydendambaev et al., 2013). Greater lipid accumulation and mobilization during seedling development occurred in the salt-sensitive variety of sunflower (*Helianthus annuus* L.) DRSH 1 than the salt-tolerant variety PSH 1962 (Gogna and Bhatla, 2020). However, lipid profiling to address membrane lipid compositional changes in salt-sensitive soybean leaves following salt treatment has not been reported.

The majority of membrane lipids in leaves are structural lipids including galactolipids (GLs) and phospholipids (PLs) (Guschina et al., 2014). Chloroplast membranes consist mainly of GLs including monogalactosyldiacylglycerol (MGDG) and digalactosyldiacylglycerol (DGDG) followed by anionic lipids comprising sulfoquinovosyldiacylglycerol (SQDG) and phosphatidylglycerol (PG) (Douce, 1974; Block et al., 1983). As the major components of photosystem II on the thylakoid membranes, MGDG and DGDG form an indispensable matrix for photosynthesis (Mizusawa and Wada, 2012), and their ratio is important in maintaining chloroplast membrane stability (Dörmann and Benning, 2002). The amounts of chloroplast lipids (MGDG, DGDG, SQDG, and PG) drastically decreased in *B. napus* leaves after cadmium stress (Nouairi et al., 2006). During drought stress, the ratio of DGDG/MGDG was elevated, mainly attributed by a fall in MGDG (Gigon et al., 2004; Torres-Franklin et al., 2007). Sodium chloride (NaCl)-induced decline in MGDG and DGDG was reported in leaves of *Thellungiella halophila*, *Sulla carnosa*, and *Sulla coronaria* (Sui and Han, 2014; Bejaoui et al., 2016).

Among the main PL classes, phosphatidic acid (PA) plays an important role in signal transduction and serves as a key intermediate in lipid metabolism (Athenstaedt and Daum, 1999). PA accumulation can be induced by biotic and abiotic stresses caused by salt, drought, cold, plant pathogens, and wounding (Tuteja and Sopory, 2008). Under duress, PA is produced through hydrolysis of the main structural PLs such as phosphatidylcholine (PC) and phosphatidylethanolamine (PE) by phospholipase D (PLD) (Pappan et al., 1998). It can also be generated by phosphorylation of diacylglycerol (DAG), which is the product of phospholipase C (PLC) hydrolysis on phosphatidylinositol (PI) (Arisz et al., 2009). PC also acts as a resource for lipid second messengers such as lysophosphatidylcholine (lysoPC), PA, and DAG (Exton, 1990). High salt and osmotic stress caused a rapid rise in PC turnover in Arabidopsis suspension−cultured cells along with an increase in phosphatidylinositol 4, 5-bisphosphate [PI(4,5)P_2_] and DAG pyrophosphate (Pical et al., 1999). Moreover, PC turnover facilitates substrates for production of chloroplast GLs (Karki et al., 2019). The chloroplast is the site of *de novo* fatty acid synthesis (FAS) with the conversion of pyruvate to acetyl-CoA and generation of carbon (C) chains 16:0, 18:0 and 18:1-acyl-carrier protein (ACP) for the assembly of PA (Hölzl and Dörmann, 2019), which can be further utilized for building chloroplast structural membranes (PG, MGDG, and DGDG) *via* the prokaryotic pathway (Boudière et al., 2014). This pathway is distinguished from the eukaryotic (Kennedy) pathway by the presence of a C chain 16 at the *sn*-2 position of the glycerol backbone (Boudière et al., 2014). For the eukaryotic pathway, 18:1-CoA esters bound to acyl-CoA binding proteins are imported into the endoplasmic reticulum (ER) and integrated to PC (Karki et al., 2019), which then undergoes desaturation to 18:2 and 18:3-CoA by acyl chain editing, dependent on the dynamic interconversion between PC and lysoPC (Bates et al., 2012). After PC desaturation, a proportion could be incorporated in the chloroplasts as DAG during GL biosynthesis (Hölzl and Dörmann, 2019; He et al., 2020).

Given the complexities in protein function upon stress treatment, proteomics represents a powerful approach to evaluate the proteome. Such analysis related to stresses such as drought, salinity, and extreme temperatures has been reported in several major crops (Ahmad et al., 2016). Furthermore, the integration of proteomics data with others from genomics, transcriptomics, or metabolomics provides wider scope in understanding biological processes (Zhang and Kuster, 2019). In soybean, proteome studies on abiotic stress have been reported on flood, drought, heat, and salinity (Das et al., 2016; Ji et al., 2016; Yin and Komatsu, 2017; Katam et al., 2020). A case in point is the integration of metabolome and proteome studies of the maize *atg12* mutant which revealed a role for ATG12 in autophagic recycling by proteome remodeling and lipid turnover (McLoughlin et al., 2018).

To explore the adaptive mechanisms of soybean in lipid signaling and remodeling of metabolism following salt stress, lipid profiling was performed on leaves of seedlings from cultivar C08 (Lam et al., 2010; Qi et al., 2014) which is a salt-sensitive variety. In our previous study, C08 seedlings showed leaf drooping at 0.5 h under high salt stress followed by recovery (Liu et al., 2019). To study if this phenomenon could be closely linked to the dynamic changes in membrane lipid composition, integrated proteomic and lipidomic analyses were conducted. To address protein function in response to salt, label-free quantitative (LFQ) proteomic analysis on salt-treated soybean seedling leaves was conducted. Our study identified putative enzymes or protein components involved in lipid regulation and provide a comprehensive understanding of lipid remodeling following salt treatment in soybean.

## Materials and Methods

### Plant Materials and Growth Conditions

Soybean *G. max* [L.] Merr., accession C08 (Lam et al., 2010) seeds were germinated in vermiculite in a greenhouse. Seedlings were transferred to 1/2 strength Hoagland nutrient solution (Hoagland and Arnon, 1950) when the primary leaves were fully expanded. Detailed growth conditions have been previously described (Liu et al., 2019). For salt treatment, 14-day-old seedlings were transferred to 1/2 strength Hoagland solution supplemented with 0.9% (w/v) NaCl. NaCl was omitted in the control. Primary leaves were harvested at 0, 0.5, 1, and 2 h after treatment.

### Lipid Extraction and Profiling

Total lipids were extracted following the protocol of Shiva et al. (2018) with minor modifications. Around six leaf punches from the primary leaf were sampled using a liquid nitrogen (N_2_) pre-cooled leaf puncher. The leaf punches were immediately immersed in 2 ml of isopropanol containing 0.01% butylated hydroxytoluene in a 20-ml vial at 75°C. The samples were heated at 75°C for 30 min to deactivate phospholipid-hydrolyzing enzymes. After cooling the samples to room temperature, 6 ml chloroform/methanol/water (30:41.5:3.5) were added, and the mixture was shaken at 100 rpm on an orbital shaker at room temperature for 24 h. To determine the dry weight after lipid extraction, the leaf punches were dried in an oven at 80°C for 72 h, cooled in an airtight desiccator, and weighed with a precision balance. The lipid content was estimated and normalized to the dry weight of the leaf punches. Five independent replicates of leaves from soybean seedlings were analyzed. Individual lipid species were denoted by the lipid class, followed by the total number of acyl Cs and the total number of C-C double bonds in the acyl chains, e.g., MGDG (34:6). The profile of membrane lipids was measured using an automated electrospray ionization-tandem mass spectrometry (Devaiah et al., 2006) at the Kansas Lipidomics Research Center.

### Protein Extraction, Digestion and Peptide Preparation

Total leaf protein was extracted following the method of Lv et al. (2013) with minor modifications. Harvested primary leaves were ground in liquid N_2_ with a mortar and pestle. The powdered sample was suspended in 2 ml of sodium dodecyl sulfate (SDS) buffer (2% [w/v] SDS, 100 mM Tris–HCl, pH 8.0, 50 mM ethylenediaminetetraacetic acid disodium salt [EDTA-Na_2_], 20 mM dithiothreitol [DTT]) with 1 mM phenylmethanesulfonyl fluoride (Sigma) and 1× protease inhibitor cocktail (Sigma). The samples were heated at 95°C for 30 min. After clarification by centrifugation at 14,000 × *g*, the protein was precipitated with three volumes of acetone at −80°C for 1 h. The protein was pelleted by centrifugation at 14,000 × *g* and then resuspended in 8 M urea 50 mM Tris–HCl, pH 8.0. The precipitation procedure was repeated once. The air-dried protein pellet was further dissolved in 8 M urea 50 mM Tris–HCl, pH 8.0 at 37°C for over 3 h. Ten microgram protein per sample was reduced with 5 mM DTT at 37°C for 1 h and trypsin-digested at 37°C overnight using Trypsin Gold, Mass Spectrometry Grade (Promega) in a trypsin:protein ratio of 1:20 (w/w). The peptide mixture was desalted using a Pierce^TM^ C-18 spin column (Thermo Fisher Scientific^TM^) and then dissolved in 0.1% (v/v) formic acid. Three biological replicates were performed.

### Orbitrap Liquid Chromatography-Tandem Mass Spectrometry (LC-MS/MS) and Quantitative Analysis

Eight hundred nanogram digested peptides from each sample was injected and analyzed using an Orbitrap Fusion^TM^ Lumos^TM^ Tribrid^TM^ Mass Spectrometer (Thermo Fisher Scientific^TM^). Nano-liquid chromatography (LC) separation of digested peptides was carried out using a LC Ultimate 3000 RSLCnano system equipped with a C-18 5 μm-pre-column (300 μm i.d. × 5 mm) and an Acclaim Pepmap RSLC nanoViper C-18 column (75 μm i.d. × 25 cm). Mobile phase A (1.9% acetonitrile and 0.1% formic acid) and mobile phase B (98% acetonitrile and 0.1% formic acid) were used in LC. The LC mobile phase gradient profile was set as follows: 50°C chamber with 3 μL min^–1^ flow rate, beginning with 100% mobile phase A for 5 min, increasing mobile phase B from 0 to 6% in 3 min, to 18% in 40 min, to 30% in 10 min, then to 80% in 7 min, and finally re-equilibrating with 100% mobile phase A for 10 min. Raw data was acquired with Xcalibur software and analyzed using the Proteome Discoverer v2.3 software (Thermo Fisher Scientific^TM^). The resulted MS/MS spectra were searched against the *G. max* reference protein database (Williams 82 a2v1) downloaded from the Phytozome portal^1^ with Proteome Discoverer internal engine SEQUEST HT. The false discovery rate (FDR) was calculated and assigned to matched peptides by Percolator (Käll et al., 2007). Peptides with an FDR-value ≤ 0.01 were selected for subsequent analysis. LFQ was performed using Proteome Discoverer comprehensive LFQ consensus workflow. Briefly, unique peptides were used for quantification, MS1 precursor abundance was estimated based on its intensity. Peptide abundance was normalized by total peptide amount and summed into protein abundances. Each time point has three biological replicates. Protein fold changes were calculated based on protein abundance using salt-treated against untreated samples, and imputation was performed with replicate based resampling. An ANOVA test was employed to test differentially expressed proteins (DEPs), *p*-value was corrected by BH method (Benjamini and Hochberg, 1995). Proteins detected in all replicates of any one of the two samples with an adjusted *p*-value ≤ 0.05 were selected as DEPs. Common and specific sets of identified proteins as well as DEPs were visualized by Venny 2.1 (Oliveros, 2007). The protein abundance identified in all three replicates was used to conduct principal component analysis (PCA), and batch effects were removed by the removeBatchEffect function of limma package (Smyth and Speed, 2003).

Gene Ontology (GO) enrichment was performed on AgriGO v2.0^2^ using GO slim annotation (Tian et al., 2017). The reference William 82 a2v1 was set as background. The Fisher exact test was employed and the *p*-value was corrected by the Yekutieli method (Yekutieli and Benjamini, 2001). GO terms were selected using a cut-off of the adjusted *p*-value ≤ 0.05 and the minimum number of mapping entries ≥5. Annotation from the Kyoto encyclopedia genes and genomes (KEGG) database was used for pathway enrichment analysis with KOBAS v2.0 (Xie et al., 2011). Protein sequences of input genes were searched against KEGG *G. max* terms by BLASTP software (Camacho et al., 2009). The hypergeometric test was further conducted to test enrichment. The matched hits were filtered by coverage ≥80% and identity ≥99%. The prediction of protein subcellular localisation was performed on ProtComp9.0^3^.

## Results

### Salt-Induced GL and PL Changes in Soybean Leaves

**FIGURE 1:**
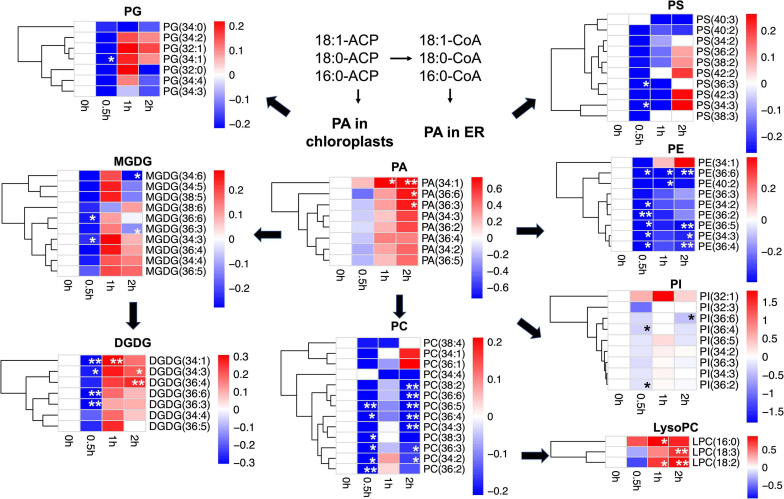
Changes in lipid acyl species under salt stress in leaves of soybean (*Glycine max* [L.] Merr.) accession C08. Heatmaps showing the log_2_-fold changes in each affected lipid acyl species at 0.5, 1, and 2 h of salt treatment. PA as lipid metabolic intermediates in the endoplasmic reticulum (ER) are derived from 16:0, 18:0-ACP, and 18:1-ACP plastidial *de novo* fatty acid synthesis. Total lipids were extracted from leaves of 14-day-old C08 seedlings grown in 1/2 strength Hoagland solution and treated with or without 0.9% (w/v) NaCl. The lipid molecular species were quantified by tandem mass spectrometry. Scale bar represents log_2_-fold changes. *, significant difference at *p* ≤ 0.05; **, significant difference at *p* ≤ 0.01 using the Student’s *t*-test. The raw data is provided in Supplementary Table 1. ACP, acyl carrier protein; DGDG, digalactosyldiacylglycerol; LysoPC, lysophosphatidylcholine; MGDG, monogalactosyldiacylglycerol; PA, phosphatidic acid; PC, phosphatidylcholine; PE, phosphatidylethanolamine; PG, phosphatidylglycerol; PI, phosphatidylinositol; PS, phosphatidylserine.

On tandem mass spectrometry (Table 1), lipid species including GLs (MGDG and DGDG), PLs (PC, PE, PG, PI, PA, and PS), and lysoPLs (lysoPC, lysoPE, and lysoPG) were identified in soybean leaf samples, and the five species most abundant in soybean leaves includes MGDG (50.0–52.8%), DGDG (16.0–18.2%), PG (11.5–12.0%), PE (7.8–8.2%), and PC (3.7–6.2%) (Supplementary Figure 1). The total content as well as several membrane lipid classes (DGDG, MGDG, PG, PC, and PE) in soybean leaves showed a rapid reduction (0.8-fold) at 0.5 h of salt treatment, which reverted to normal at 1 h (Table 1). LysoPC increased by 1.4-fold at 1 h and 1.7-fold at 2 h of salt treatment (Table 1). For PA, a critical intermediate in lipid biosynthesis in both the chloroplasts and the ER, PA (34:1) accumulated at 1 and 2 h, and PA (36:3) and PA (36:6) were elevated at 2 h (Figure 1 and Supplementary Table 1). For MGDG, DGDG, and PG, forming major components of chloroplast membranes primarily derived from PA in the plastids, abundant acyl species were MGDG (36:6), DGDG (34:3), DGDG (36:6), PG (32:1), and PG (34:2) (Supplementary Table 1). After salt treatment, MGDG (36:6), DGDG (36:6), and DGDG (34:3) significantly dipped at 0.5 h, but reverted to normal at 1 h (Figure 1 and Supplementary Table 1). Of the three, only DGDG (34:3) rose markedly at 2 h after salt treatment (Figure 1 and Supplementary Table 1). Moreover, the ratio of DGDG/MGDG, important in maintaining chloroplast membrane stability, increased at 1 and 2 h (Supplementary Figure 2). Amongst the PG acyl species, only PG (34:1) showed significant decrease at 0.5 h of salt treatment (Figure 1 and Supplementary Table 1).

**TABLE 1:** Changes in major lipid classes of soybean C08 leaves under salt treatment.

Lipid class	Untreated control				Salt treatment			Fold change (stress vs. control)		
	0 h	0.5 h	1 h	2 h	0.5 h	1 h	2 h	0.5 h	1 h	2 h
DGDG	7.24 ± 0.55	7.10 ± 0.35	6.49 ± 0.68	6.92 ± 0.52	5.72 ± 0.75**	7.44 ± 1.49	7.49 ± 0.40	**0.81**	1.15	1.08
MGDG	21.40 ± 1.77	21.19 ± 1.44	20.45 ± 2.50	21.47 ± 1.67	17.81 ± 2.38*	22.16 ± 4.76	21.41 ± 0.94	**0.84**	1.08	1.00
PG	5.14 ± 0.59	4.71 ± 0.36	4.63 ± 0.49	4.66 ± 0.43	3.90 ± 0.59*	5.16 ± 0.99	4.84 ± 0.21	**0.83**	1.11	1.04
PC	3.49 ± 0.29	3.18 ± 0.23	3.52 ± 0.18	3.31 ± 0.25	2.650 ± 0.31*	3.49 ± 0.92	2.89 ± 0.10	**0.83**	0.99	0.87
PE	2.11 ± 0.22	1.96 ± 0.33	2.49 ± 0.28	1.86 ± 0.26	1.47 ± 0.20*	2.03 ± 0.51	1.53 ± 0.22	**0.75**	0.82	0.83
PI	1.14 ± 0.17	0.99 ± 0.09	1.01 ± 0.02	0.96 ± 0.05	0.86 ± 0.14	1.07 ± 0.23	0.97 ± 0.07	0.87	1.06	1.01
PS	0.22 ± 0.03	0.18 ± 0.06	0.20 ± 0.02	0.16 ± 0.03	0.14 ± 0.02	0.18 ± 0.04	0.17 ± 0.03	0.75	0.92	1.03
PA	2.00 ± 0.55	1.64 ± 0.49	1.57 ± 0.24	1.22 ± 0.26	1.44 ± 0.40	1.88 ± 0.50	1.71 ± 0.44	0.88	1.20	1.40
LysoPC	0.05 ± 0.01	0.04 ± 0.01	0.04 ± 0.01	0.03 ± 0.01	0.03 ± 0.01	0.06 ± 0.01*	0.05 ± 0.01**	0.81	**1.42**	**1.74**
LysoPG	0.03 ± 0.01	0.02 ± 0.01	0.04 ± 0.02	0.03 ± 0.02	0.02 ± 0.01	0.03 ± 0.01	0.05 ± 0.01	0.97	0.80	1.73
LysoPE	0.01 ± 0.01	0.02 ± 0.01	0.01 ± 0.00	0.01 ± 0.01	0.01 ± 0.01	0.02 ± 0.01	0.01 ± 0.01	0.59	1.68	1.01
In total	42.81 ± 3.56	41.02 ± 2.68	40.44 ± 3.91	40.63 ± 3.91	34.00 ± 4.21*	43.51 ± 9.03	41.13 ± 1.77	0.83	1.08	1.01

Values are means ± SD (nmol/mg dry weight; n = 5). Significant differences between control and salt treatment are bolded.*, Significant difference at p ≤ 0.05.**, Significant difference at p ≤ 0.01 using the Student’s t-test.

When PC and PE, the major PLs in the cell membranes formed from PA in the ER were analyzed, it was observed that PC acyl species including PC (36:3), PC (36:4), PC (36:5), and PC (34:2) were lower at 0.5 h after salt treatment, recovered at 1 h, and dropped again at 2 h (Figure 1 and Supplementary Table 1). In contrast, all detected molecular species of lysoPC, including lysoPC (16:0), lysoPC (18:2), and lysoPC (18:3) were elevated only at 1 or 2 h (Figure 1 and Supplementary Table 1). Similar to PC, most molecular species of PE declined after 0.5 h and again at 2 h, with PE (36:6) reduced at all the time points (Figure 1 and Supplementary Table 1). The ratio of PC/PE for 34:3, 34:2, 36:6, 36:5, and 36:2 increased at 1 h (Supplementary Table 2). Also, the PI species 36:2 and 36:4 were lower at 0.5 h and 36:6 declined after 2 h of salt treatment (Figure 1 and Supplementary Table 1). Moreover, the unsaturation levels of certain lipid species decreased, primarily in MGDG, PC, PE, PS, and PA at 1 and 2 h after salt treatment (Supplementary Table 3). These changes in various membrane lipid molecular species indicate that salt stress altered membrane lipids dynamically in C08 leaves.

### Proteomic Analysis of C08 Leaves Under Salt Stress

**FIGURE 2:**
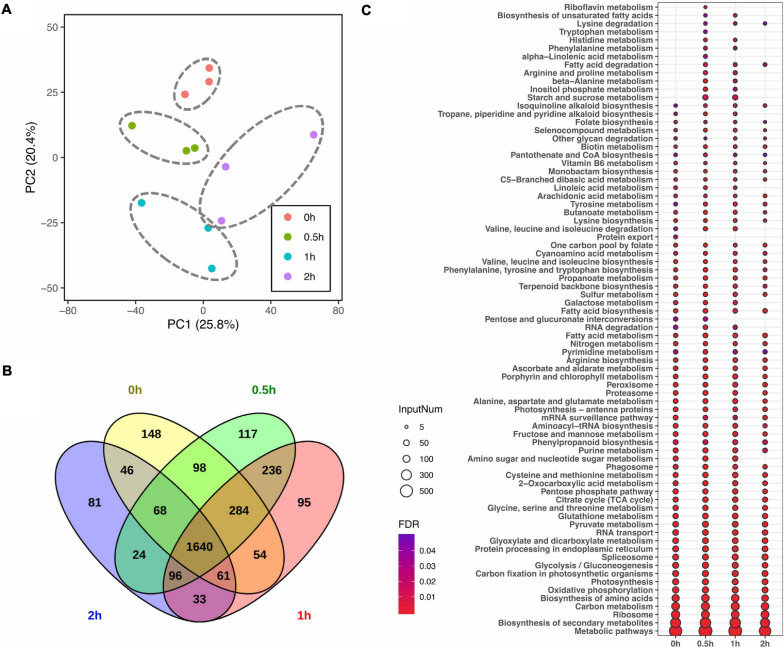
Impact of salt treatment for 0, 0.5, 1, and 2 h on leaf proteome of soybean (*Glycine max* [L.] Merr.) accession C08. Total leaf protein was extracted and analyzed by tandem mass spectrometry from 14-day-old C08 seedlings grown in 1/2 strength Hoagland solution and treated with or without 0.9% (w/v) NaCl. **(A)** PCA of proteome profiles. Normalized protein abundance was Log_2_ transformed and used as input data, centring and scaling was performed prior than calculating principle components. The first and second principal components were visualized in dot plot, and the variation was labeled in the brackets. **(B)** Venn diagram of identified proteins in each time point. **(C)** Kyoto Encyclopedia of Genes and Genomes (KEGG) analysis of identified proteins from each time point. The color gradient represents the value of a false discovery rate, with a cut-off ≤0.05. The size of each circle represents the input number of proteins in the enriched pathway. The protein lists of enriched pathways are provided in Supplementary Table 4.

Given that lipid turnover in soybean leaves is facilitated by a series of metabolic enzymes, regulatory proteins and their related transporters, to identify the molecular mechanisms in lipid modulation, proteome profiling was performed in parallel to lipid profiling. In total, 2,399, 2,563, 2,499, and 2,049 master proteins were detected at 0, 0.5, 1, and 2 h, respectively, after salt treatment (Table 2). When the entire proteome was analyzed by PCA, clear differences among time points were observed with three biological replicates of each time point tending to cluster together (Figure 2A). Venn diagram analysis of identified proteins from all time points showed that 1,640 master proteins were common, and 148, 117, 95, and 81 unique proteins exist at 0, 0.5, 1, and 2 h, respectively (Figure 2B).

**TABLE 2:** Summary of soybean C08 leaf proteome before and after salt treatment.

Salt treatment (h)	Samples (bioreplicates)	PSM*	Peptide groups	Identified master proteins
Untreated control	1	13,173	7,229	
	2	19,980	14,419	2,399
	3	19,885	14,155	
0.5	1	19,732	14,406	
	2	20,013	14,224	2,563
	3	13,517	8,620	
1.0	1	14,382	10,691	
	2	19,755	13,990	2,499
	3	14,326	8,494	
2.0	1	14,125	8,095	
	2	12,131	7,907	2,049
	3	11,194	6,628	

*PSM, peptide spectrum matches.

Pathway enrichment analysis was conducted by identifying KEGG pathways with all identified proteins with or without salt treatment. In comparison to 0 h, 11 and 7 metabolic or signaling pathways were enriched at 0.5 and 1 h, including pathways associated with amino acid (aa) biosynthesis and degradation, starch and sucrose metabolism, fatty acid (FA) metabolism and secondary metabolite biosynthesis pathways (Figure 2C and Supplementary Table 4). Furthermore, protein export was specially enriched at 0 h. The number of proteins enriched in biotin metabolism and FA biosynthesis increased after salt stress, while those associated with nucleotide excision repair and RNA degradation pathway declined (Figure 2C and Supplementary Table 4).

**FIGURE 3:**
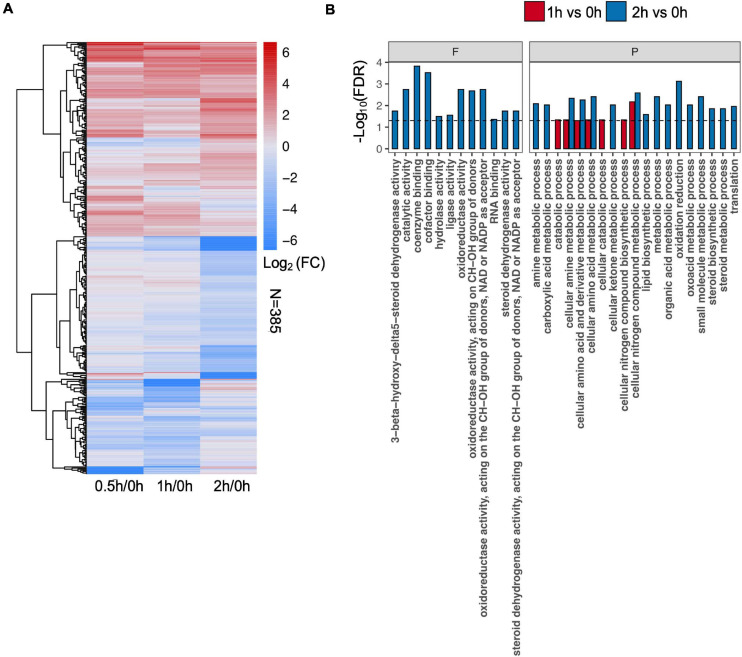
Effect of salt treatment on the soybean (*Glycine max* [L.] Merr.) accession C08 leaf proteome. **(A)** Heatmap displays the log_2_-fold changes in abundance of significant differentially expressed proteins (DEPs) (stress vs. normal control), adjusted *p*-value ≤ 0.05. The union set was used to generate the plot. **(B)** Gene ontology (GO) enrichment of DEPs at each time point compared to 0 h sample. Three categories of GO terms including “cellular component,” “biological process” (P), and “molecular function” (F) were used, only items in the last two were found enriched. Dashed line indicates FDR = 0.05. The protein lists of enriched items are provided in Supplementary Table 6.

To identify differential protein abundance at each time point in C08 leaves under salt stress, LFQ analysis was carried out and the fold changes of protein abundance under stress vs. the control (i.e., 0.5/0, 1/0, and 2/0 h) calculated. The results revealed that 120 (81 upregulated), 181 (78 upregulated), and 206 (91 upregulated) DEPs were identified after 0.5, 1, and 2 h, respectively, of salt treatment in comparison to the untreated sample (Figure 3A and Supplementary Table 5). GO analysis of DEPs from 0.5, 1, and 2 h is shown in Figure 3B and Supplementary Table 6. In the biological process category, for enrichment at 1 h DEPs were associated mainly with amino acid metabolism, nitrogen compound biosynthesis, while DEPs at 2 h clustered more in steroid metabolism, lipid biosynthesis, oxidation reduction, and translation process (Figure 3B). Some stress responsive proteins including dehydrin and ROS scavenging enzyme glutathione S-transferase (GST) and heat shock proteins (HSPs) which accumulated after salt treatment corresponded with their induced transcription (Supplementary Table 7). Moreover, a major proportion DEPs was predicted to localize in the chloroplasts, mitochondria, nucleus, and cytoplasm after salt treatment (Supplementary Figure 3).

### Alteration of *de novo* FA and JA Biosynthetic Enzymes in the Chloroplasts During Salt Stress

**FIGURE 4:**
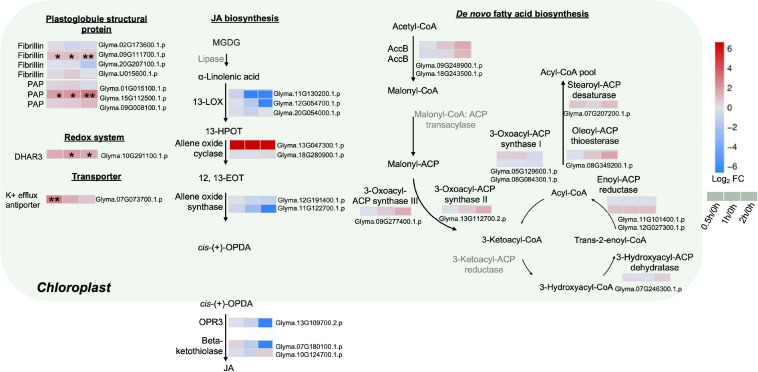
Proteins with differential abundance related to various metabolic pathways in the chloroplasts and peroxisomes during salt stress in leaves of soybean (*Glycine max* [L.] Merr.) accession C08. Each significant differentially expressed protein is presented as heatmaps with shades of red or blue according to the scale bar. Scale bar indicates log_2_-fold changes of protein abundance (stress vs. control) with an adjusted *p*-value ≤ 0.05. *, significant difference at *p* ≤ 0.05; **, significant difference at *p* ≤ 0.01 using ANOVA test. For tandem mass spectrometry, the total leaf protein was extracted from leaves of 14-day-old C08 seedlings grown in 1/2 strength Hoagland solution and treated with or without 0.9% (w/v) NaCl. Differential protein abundances were calculated by label-free quantification of MS peptide signals. AccB, biotin carboxyl carrier protein of acetyl-CoA carboxylase; ACP, acyl carrier protein; DHAR3, dehydroascorbate reductase 3; 12, 13-EOT, 12, 13-epoxyoctadeca-9, 11, 15-trienoic acid; 13-HPOT, 13 hydroperoxyoctadeca-9, 11, 15-trienoic acid; JA, jasmonic acid; 13-LOX, 13-lipoxygenases; OPR3, 12-oxophytodienoate reductase 3; PAP, plastid lipid-associated protein.

To further explore the possible mechanisms underlining redistribution of membrane lipids at the early stages of salt treatment, various proteins from the chloroplasts associated with FA and jasmonic acid (JA) biosyntheses were analyzed. Major enzymes related to *de novo* FAS were detected with no obvious changes, including the rate-limiting enzyme acetyl-CoA carboxylase (ACC), 3-oxoacyl-ACP synthase I, II, III, 3-hydroxyacyl-ACP dehydratase, enoyl-ACP reductase, oleoyl-ACP thioesterase, and stearoyl-ACP desaturase (Figure 4 and Supplementary Table 8).

The enzymes related to the biosynthesis of JA, a fatty acid-derived phytohormone regulating abiotic stress responses in plants, were investigated (Figure 4 and Supplementary Table 8). Most key enzymes chloroplast-localized 13S-lipoxygenase (13-LOX), allene oxide cyclase (AOC), allene oxide synthase (AOS), 12-oxophytodienoate reductase 3 (OPR3) as well as the peroxisome-localized β-ketothiolase were identified with no obvious changes in our results (Figure 4 and Supplementary Table 8). Only one AOC (Glyma.13G047300.1.p) was detected only after salt treatment (Figure 4 and Supplementary Table 8).

As stress triggers membrane lipid degradation and the resultant polyunsaturated fatty acids (PUFAs) can be stored as triacylglycerol (TAG) in the plastoglobules (lipid bodies in the chloroplasts), it was not surprising that plastoglobule structural proteins, fibrillin and plastid-lipid-associated protein (PAP) (homologs of the fibrillin family) increased at 0.5, 1 and 2 h of salt treatment (Figure 4 and Supplementary Table 8). Fibrillin (Glyma.09G111700.1.p) and PAP (Glyma.15G112500.1.p) were induced continuously from 0.5 to 2 h of salt treatment (Figure 4 and Supplementary Table 8). Moreover, other plastidial proteins were also identified. The enzymatic antioxidant dehydroascorbate reductase 3 (DHR3) was elevated by three-fold at 2 h. The K^+^ efflux antiporter, which facilitates endosomal pH homeostasis and salt tolerance, increased at 0.5 h (Figure 4 and Supplementary Table 8).

### Mitochondrial TCA Cycle and Cytosolic Glycolysis Enhanced During Salt Stress

**FIGURE 5:**
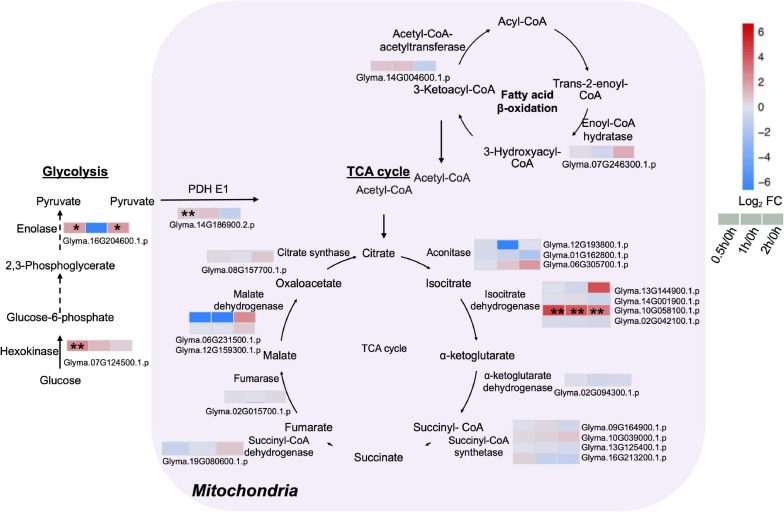
Proteins with differential abundance involved in the tricarboxylic acid (TCA) cycle, glycolysis and fatty acid β-oxidation pathway during salt stress in leaves of soybean (*Glycine max* [L.] Merr.) accession C08. Each significant differentially expressed protein is presented as heatmaps with shades of red or blue according to the scale bar. Scale bar indicates log_2_-fold changes of protein abundance (stress vs. control) with an adjusted *p*-value ≤ 0.05. *, significant difference at *p* ≤ 0.05; **, significant difference at *p* ≤ 0.01 using ANOVA test. Black arrow, reaction between two intermediates; dashed arrow, several steps in the reaction. For tandem mass spectrometry, the total leaf protein was extracted from leaves of 14-day-old C08 seedlings grown in 1/2 strength Hoagland solution and treated with or without 0.9% (w/v) NaCl. Differential protein abundances were calculated by label-free quantification of MS peptide signals. PDH E1, pyruvate dehydrogenase E1 component.

Given that energy production processes such as glycolysis and TCA cycle are known to respond to early salt exposure, analysis on proteins related to these two pathways were conducted. The major enzymes of the TCA cycle including citrate synthase, malate dehydrogenase, succinate dehydrogenase, succinate synthetase, α-ketoglutarate, and isocitrate dehydrogenase were detected (Figure 5 and Supplementary Table 8). An isocitrate dehydrogenase (Glyma.10G058100.1.p) was upregulated at least 7.8-fold during salt treatment (Figure 5 and Supplementary Table 8). Some enzymes of the cytoplasmic glycolysis pathway also increased. A hexokinase (Glyma.07G124500.1.p) rose five-fold at 0.5 h, while an enolase (Glyma.16G204600.1.p) increased four-fold at 0.5 and 2 h after salt treatment (Figure 5 and Supplementary Table 8). Proteome data indicated a three-fold increase in pyruvate dehydrogenase (PDH) complex E1 (Glyma.14G186900.2.p) at 0.5 h of salt treatment (Figure 5 and Supplementary Table 8). PDH is a vital metabolic enzyme that links glycolysis to the TCA cycle by converting pyruvate to acetyl-CoA, and increasing the influx of acetyl-CoA from glycolysis into the TCA cycle. These results indicate that in C08 leaves, the TCA cycle and the glycolysis pathway were enhanced by salt treatment.

### PI Signaling and PL Metabolism in the ER Are Affected by Salt Treatment

**FIGURE 6:**
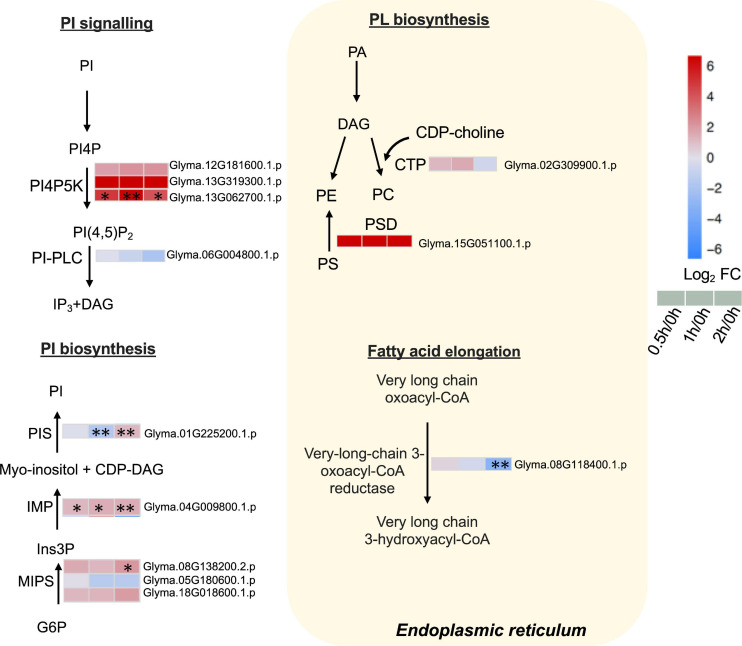
Proteins with differential abundance involved in phospholipid metabolic pathways during salt stress in leaves of soybean (*Glycine max* [L.] Merr.) accession C08. Each significant differentially expressed protein is presented as heatmaps with shades of red or blue according to the scale bar. Scale bar indicates log_2_-fold changes of protein abundance (stress vs. control) with an adjusted *p*-value ≤ 0.05. ^∗^, significant difference at *p* ≤ 0.05; ^∗∗^, significant difference at *p* ≤ 0.01 using ANOVA test. For tandem mass spectrometry, the total leaf protein was extracted from leaves of 14-day-old C08 seedlings grown in 1/2 strength Hoagland solution and treated with or without 0.9% (w/v) NaCl. Differential protein abundances were calculated by label-free quantification of MS peptide signals. CDP-choline, cytidine diphosphate choline; CDP-DAG, cytidine diphosphate diacylglycerol; PA, phosphatidic; CTP, choline-phosphate cytidylyltransferase; DAG, diacylglycerol; G6P, glucose 6-phosphate; Ins3P, *myo*–inositol–3–phosphate; IMP, inositol monophosphatase; MIPS, *myo*-inositol-1-phosphate synthase; PA, phosphatidic acid; PC, phosphatidylcholine; PE, phosphatidylethanolamine; PI, phosphatidylinositol; PI(4,5)P_2_, phosphatidylinositol 4, 5-bisphosphate; PI4P, phosphatidylinositol 4-phosphate; PI4PK5, phosphatidylinositol-4-phosphate 5-kinase; PI-PLC, phosphoinositide phospholipase C; PIS, phosphatidylinositol synthase; PS, phosphatidylserine; PS decarboxylase, phosphatidylserine decarboxylase.

Our proteome data detected three phosphatidylinositol-4-phosphate 5-kinases (PI4P5Ks), of which one (Glyma.13G062700.1.p) was elevated continuously from 0.5 to 2 h (Figure 6 and Supplementary Table 8). Phosphatidylinositol phospholipase C (PI-PLC), responsible for converting PI (4,5)P_2_ to inositol 1,4,5-trisphosphate (IP_3_) and DAG, was detected but not altered in our dataset (Figure 6 and Supplementary Table 8).

Furthermore, the key enzymes related to PI biosynthesis accumulated after salt treatment. The ER-localized PI synthase (PIS) decreased by 0.3-fold at 1 h and increased by 2.3-fold at 2 h (Figure 6 and Supplementary Table 8). The *myo*-inositol-3-phosphate synthase (MIPS, Glyma.08G138200.2.p) was induced 4.4-fold at 2 h and inositol monophosphatase (IMP) (Glyma.04G009800.1.p) was upregulated from 0.5 to 2 h salt treatment (Figure 6 and Supplementary Table 8). Moreover, when other enzymes related membrane PL biosynthesis in the ER were examined, the results revealed that choline-phosphate cytidylyltransferase (CTP) essential in PC formation was not altered after salt treatment (Figure 6 and Supplementary Table 8). PS decarboxylase (PSD) (Glyma.15G051100.1.p) that converts PS to PE, was only identified after salt treatment (Figure 6 and Supplementary Table 8). The very-long-chain 3-oxoacyl-CoA reductase (KCR), required for the elongation of fatty acids, had decreased at 2 h of salt treatment (Figure 6 and Supplementary Table 8).

## Discussion

### Salt Stress Affects Membrane Lipid Composition

Earlier studies have shown the repercussion of salt stress on metabolites (Li et al., 2017), carbohydrate pool rebalancing aa metabolism (Zhang et al., 2017) and protein turnover (Kosová et al., 2013) as well as the maintenance of primary N homeostasis in various crops (Wang et al., 2012). Here we report on how high salt impacted membrane lipid composition and reveal a correlation in lipid metabolism with the remodulation of C and N pools upon salt treatment. Integration of lipidome and proteome data in a time series better facilitated an understanding on lipid turnover and protein alteration under salt stress. Salinity causes cell membrane breakdown and electrolyte leakage in soybean (Phang et al., 2008) and affects lipid composition (Shoemaker and Vanderlick, 2002). Leaf drooping was observed upon 30 min exposure to 0.9% salt solution, and recovery was evident at 1 h (Liu et al., 2019). Consistent with this, our lipid profiling of salt-treated soybean leaves showed a sharp decrease in total lipid content at 0.5 h of salt treatment which returned to normal at 1 h. The reduction and recovery of total lipids indicate that lipid composition could be rebalanced within a short period of time upon salt treatment of C08 leaves.

Our results showed a reduction in the major MGDG acyl species and most DGDG acyl species at 0.5 h of salt stress (Figure 1 and Supplementary Table 1), consistent with salt-triggering GL reduction in leaves of rice, *Cucumis sativus* and *Vigna unguiculata* (De Paula et al., 1990; Yamane et al., 2004; Shu et al., 2012). Decreases in DGDG and MGDG are known to adversely affect photosynthetic membranes and membrane protein activity in the thylakoids (Simidjiev et al., 2000), and reduction of MGDG is a typical response to osmotic stress arising from salinity, drought or freezing in Arabidopsis (Welti et al., 2002; Gigon et al., 2004). Besides, an increased ratio in DGDG/MGDG at 1 and 2 h of salt treatment (Supplementary Figure 1) is consistent with a higher ratio in DGDG/MGDG in cowpea leaves after drought stress enhanced thylakoid stability and maintained the bilayer structure through the synthesis of stabilizing DGDG lipid species (Torres-Franklin et al., 2007). Therefore, a reduction in the major species of DGDG and MGDG and an increase in the DGDG/MGDG ratio of salt treatment may be a consequence of the disruption in chloroplast membrane stability and photosystem function at the onset of salt treatment.

Most PC and PE species declined at 0.5 h and again at 2 h after salt treatment (Figure 1 and Supplementary Table 1), consistent with a degradation in major acyl species of PC and PE under drought stress in Arabidopsis leaves and winter wheat seedlings (Gigon et al., 2004; Wang et al., 2020). PC and PE are the major PLs in the structural membranes, and PC is known to be involved in the adaption to abiotic stresses (Tasseva et al., 2004). Increase in PC species following salt stress has been reported in salt-tolerant plants including *Catharanthus roseus* and *Mesembryanthemum crystallinum* (Elkahoui et al., 2004; Barkla et al., 2018) while a decrease in PC species occurs in the salt-sensitive plants such as oats and wheat (Norberg and Liljenberg, 1991; Magdy et al., 1994).

### Salt Stress Elevated Plastidial Proteins Related to Plastoglobules and JA Biosynthesis

Our results showed that AOC, a key enzyme in JA production, accumulated at 0.5, 1, and 2 h after salt treatment (Figure 4 and Supplementary Table 8). JA, a major phytohormone regulating abiotic stress responses, is important in early responses to osmotic and salt stress (Fujita et al., 2006; Chen et al., 2017) and its biosynthesis was triggered by salt in Arabidopsis (Ishiguro et al., 2001; Wang et al., 2015, 2018). AOC affects the rate of flux through the JA biosynthetic pathway (Yoeun et al., 2018). Overexpression of *TaAOC1* conferred salt tolerance in wheat (Zhao et al., 2014). Using 2-DE and MALDI-TOF-MS/MS, higher AOC was reported in *Medicago sativa* following early salt stress (Xiong et al., 2017) while upregulated AtAOC2 protein abundance was reported in Arabidopsis under salt stress using 2-DE and iTRAQ LC-MS/MS (Pang et al., 2010). Our results lead support to lead support to an enhancement of JA production following salt treatment.

The upregulation of plastoglobule structural proteins was evident at the early stages of salt treatment (Figure 4 and Supplementary Table 8). Plastoglobules are known to play an essential role in lipid remodeling of the thylakoids (Rottet et al., 2015) and increase in size and number following FA accumulation under stress conditions (Bréhélin and Kessler, 2008; Rottet et al., 2015). They have been reported to be the site for the initiation of chloroplast stress-related JA biosynthesis (Hölzl and Dörmann, 2019). The accumulation of fibrillin FBN1-2 was found to affect JA biosynthesis under photosynthetic stress (Youssef et al., 2010). In Arabidopsis, FIBRILLIN2 protects photosystem II against abiotic stress by interacting with AOS and LOX of JA biosynthesis (Torres-Romero et al., 2020). Our results suggest that the breakdown of chloroplast membrane lipids (MGDG and DGDG) would lead to the production of PUFAs (Boudière et al., 2014), which act as substrates for JA biosynthesis at the onset of salt stress (Figure 4 and Supplementary Table 8).

### Enhanced Mitochondrial TCA Cycle and Cytosolic Glycolysis at the Onset of Salt Treatment

Some enzymes involved in the TCA cycle and glycolysis pathway increased after salt treatment (Figure 5 and Supplementary Table 8), consistent with other proteome studies reported that major metabolic pathways in plants involved in energy generation (glycolysis, pyruvate decarboxylation and TCA cycle) induced by salt (Hossain et al., 2013). The TCA cycle is a crucial component of respiratory metabolism and an increased respiration after salinity exposure represents a short-term adjustment in demand for energy consumption (Bloom and Epstein, 1984). When salt-sensitive and salt-tolerant barley cultivars were subjected to short-term salinity exposure, both accumulated TCA cycle intermediates in the root elongation zone due to increased energy demand for cell division (Shelden et al., 2016). Pyruvate accumulated in both salt-sensitive and salt-tolerant soybean varieties under salt stress (Zhang et al., 2016). Pyruvate, the final product of the glycolysis pathway, can be converted to acetyl-CoA by the PDH complex and enter into the TCA cycle (Schertl and Braun, 2014). A higher level of certain enzymes in the glycolysis pathway and the TCA cycle during salt treatment (Figure 5 and Supplementary Table 8) enables accelerated respiration (Bloom and Epstein, 1984), to accommodate increased FAS and salt stress adaption in C08 leaves.

### PI Signaling and Biosynthesis Triggered at the Onset of Salt Stress

Phosphoinositides, vital lipid signaling molecules in response to salt, are derived from PI by the action of lipid kinases and phosphatases (Heilmann and Heilmann, 2015). PI4P5K catalyzes phosphatidylinositol 4-phosphate (PI4P) and generate PI(4,5)P_2_ (Delage et al., 2013). Our data revealed an accumulation of PI4P5K at the initiation of salt treatment (Figure 6 and Supplementary Table 8). PI(4,5)P_2_ is a secondary messenger that functions in recruitment of signaling complexes to specific membrane locations (Martin, 1998). PI(4,5)P_2_ is synthesized by PI4P5K which is the flux-limiting step in plant phosphoinositide metabolism (Yang et al., 2007). PI4P5K could interact with PI(4,5)P_2_ and channel them toward different targets (Heilmann and Heilmann, 2015). While in response to salt stress, PI(4,5)P_2_ levels rapidly (<30 min) rose in rice leaves (Darwish et al., 2009). In Arabidopsis, the expression of PI4P5K was induced by salt stress within 1 h (Mikami et al., 1998). Our results also support the early response of PI4P5K following salt stress. Increase in PI4P5K will supply PI(4,5)P_2_ as substrates for phospholipases (Stenzel et al., 2008).

Enzymes involved in PI biosynthesis including IMP, MIPS, and PIS accumulated during salt treatment (Figure 6 and Supplementary Table 8). MIPS, the first step of *myo*-inositol generation, coverts glucose 6-phosphate (G6P) to *myo*−inositol−3−phosphate (Ins3P), which further forms free *myo*-inositol by IMP (Loewus and Murthy, 2000). MIPS−produced *myo*−inositol plays a pivotal role in protective mechanisms in salt−tolerant plant species in *M. crystallinum* (Nelson et al., 1998). *PcINO1* encoding MIPS has been reported as a salt response protein in halophytic wild rice *Porteresia coarctata* Tateoka (Dastidar et al., 2006).

The overexpression of maize *ZmPIS* enhanced drought tolerance by elevating DGDG and MGDG contents and ABA synthesis in maize (Liu et al., 2013). Our results suggest that the increase of PI biosynthesis may subsequently accelerate the formation of products such as PI(4,5)P_2_, DAG, InsP_3_, and PA, boosting PI signaling cascades at the onset of salt stress.

## Conclusion

In conclusion, our study shows the impact of short-term salt stress on lipid metabolism in a salt-sensitive soybean germplasm. Lipid remodeling was found to occur as early as 0.5 h of salt treatment coincident with the triggering of the PI signaling pathway. Membrane lipid recycling was observed to be related to an acceleration of plastidial JA biosynthesis and PI production. The TCA cycle and glycolysis pathways were activated for energy metabolism. In summary, salt stress caused an alteration in lipid composition and rapidly activated related protein responses in C08 leaves.

## Data Availability Statement

The raw values obtained from the lipidomic data sets are available in Supplementary Table 1 (associated with Table 1 and Figure 1). Gene expression in Supplementary Table 7 was based on data retrieved from our previous study (Liu et al., 2019; NCBI SRA database with accession number SRP132150). The raw data and msf files for mass spectrometry proteomics data have been deposited to the ProteomeXchange Consortium via the PRIDE (https://www.ebi.ac.uk/pride/) partner repository with the dataset identifier PXD023092. The proteomics raw data are available in Supplementary Table 9. If any data sets are unavailable through the links stated above, they can be obtained from the corresponding author on the request.

## Author Contributions

M-LC and H-ML planned and designed the research. AL and ZW performed the experiments. ZX analyzed the data. AL, ZX and M-LC wrote the manuscript. All authors have read and approved the manuscript.

## Conflict of Interest

The authors declare that the research was conducted in the absence of any commercial or financial relationships that could be construed as a potential conflict of interest.

## Footnotes

1 https://phytozome.jgi.doe.gov/pz/portal.html

2 http://systemsbiology.cau.edu.cn/agriGOv2/

3 http://www.softberry.com/berry.phtml?topic=protcomppl&group=programs&subgroup=proloc

## References

[B1] Ahmad P. Abdel Latef A. A. H. Rasool S. Akram N. A. Ashraf M. Gucel S. (2016). Role of proteomics in crop stress tolerance. *Front. Plant Sci.* 7:1336. 10.3389/fpls.2016.01336 27660631 PMC5014855

[B2] Arisz S. A. Testerink C. Munnik T. (2009). Plant PA signaling via diacylglycerol kinase. *Biochim. Biophys. Acta Mol. Cell Biol. Lipids* 1791 869–875. 10.1016/j.bbalip.2009.04.006 19394438

[B3] Athenstaedt K. Daum G. (1999). Phosphatidic acid, a key intermediate in lipid metabolism. *Eur. J. Biochem.* 266 1–16. 10.1046/j.1432-1327.1999.00822.x 10542045

[B4] Barkla B. J. Garibay-Hernández A. Melzer M. Rupasinghe T. W. T. Roessner U. (2018). Single cell-type analysis of cellular lipid remodelling in response to salinity in the epidermal bladder cells of the model halophyte *Mesembryanthemum crystallinum*. *Plant. Cell. Environ.* 41 2390–2403. 10.1111/pce.13352 29813189

[B5] Barrero-Sicilia C. Silvestre S. Haslam R. P. Michaelson L. V. (2017). Lipid remodelling: unravelling the response to cold stress in arabidopsis and its extremophile relative *Eutrema salsugineum*. *Plant Sci.* 263 194–200. 10.1016/j.plantsci.2017.07.017 28818375 PMC5567406

[B6] Bates P. D. Fatihi A. Snapp A. R. Carlsson A. S. Browse J. Lu C. (2012). Acyl editing and headgroup exchange are the major mechanisms that direct polyunsaturated fatty acid flux into triacylglycerols. *Plant Physiol.* 160 1530–1539. 10.1104/pp.112.204438 22932756 PMC3490606

[B7] Bejaoui F. Salas J. J. Nouairi I. Smaoui A. Abdelly C. Martínez-Force E. (2016). Changes in chloroplast lipid contents and chloroplast ultrastructure in *Sulla carnosa* and *Sulla coronaria* leaves under salt stress. *J. Plant Physiol.* 198 32–38. 10.1016/j.jplph.2016.03.018 27131842

[B8] Benjamini Y. Hochberg Y. (1995). Controlling the false discovery rate: a practical and powerful approach to multiple testing. *J. R. Stat. Soc. Ser. B.* 57 289–300. 10.1111/j.2517-6161.1995.tb02031.x

[B9] Block M. A. Dorne A. J. Joyard J. Douce R. (1983). Preparation and characterization of membrane fractions enriched in outer and inner envelope membranes from spinach chloroplasts. *II. Biochemical characterization*. *J. Biol. Chem.* 258 13281–13286. 6630230

[B10] Bloom A. Epstein E. (1984). Varietal differences in salt-induced respiration in barley. *Plant Sci. Lett.* 35 1–3. 10.1016/0304-4211(84)90149-4

[B11] Boudière L. Michaud M. Petroutsos D. Rébeillé F. Falconet D. Bastien O. (2014). Glycerolipids in photosynthesis: composition, synthesis and trafficking. *Biochim. Biophys. Acta Bioenerg.* 1837 470–480. 10.1016/j.bbabio.2013.09.007 24051056

[B12] Bréhélin C. Kessler F. (2008). The plastoglobule: a bag full of lipid biochemistry tricks. *Photochem. Photobiol.* 84 1388–1394. 10.1111/j.1751-1097.2008.00459.x 19067960

[B13] Camacho C. Coulouris G. Avagyan V. Ma N. Papadopoulos J. Bealer K. (2009). BLAST+: architecture and applications. *BMC Bioinformatics* 10 1–9. 10.1186/1471-2105-10-421 20003500 PMC2803857

[B14] Chalbi N. Hessini K. Gandour M. Mohamed S. Smaoui A. Abdelly C. (2013). Are changes in membrane lipids and fatty acid composition related to salt-stress resistance in wild and cultivated barley? *J. Plant Nutr. Soil Sci.* 176 138–147. 10.1002/jpln.201100413

[B15] Chen J. Zhang J. Hu J. Xiong W. Du C. Lu M. (2017). Integrated regulatory network reveals the early salt tolerance mechanism of *Populus euphratica*. *Sci. Rep.* 7:6769. 10.1038/s41598-017-05240-0 28754917 PMC5533726

[B16] Darwish E. Testerink C. Khalil M. El-Shihy O. Munnik T. (2009). Phospholipid signaling responses in salt-stressed rice leaves. *Plant Cell. Physiol.* 50 986–997. 10.1093/pcp/pcp051 19369274 PMC2682722

[B17] Das A. Eldakak M. Paudel B. Kim D. W. Hemmati H. Basu C. (2016). Leaf proteome analysis reveals prospective drought and heat stress response mechanisms in soybean. *Biomed. Res. Int.* 2016:6021047. 10.1155/2016/6021047 27034942 PMC4808539

[B18] Dastidar K. G. Maitra S. Goswami L. Roy D. Das K. P. Majumder A. L. (2006). An insight into the molecular basis of salt tolerance of L-*myo*-inositol 1-P synthase (PcINO1) from *Porteresia coarctata* (Roxb.) Tateoka, a halophytic wild rice. *Plant Physiol.* 140 1279–1296. 10.1104/pp.105.075150 16500989 PMC1435794

[B19] De Paula F. M. Thi A. T. P. De Silva J. V. Justin A. M. Demandre C. Mazliak P. (1990). Effects of water stress on the molecular species composition of polar lipids from *Vigna unguiculata* L. leaves. *Plant Sci.* 66 185–193. 10.1016/0168-9452(90)90203-Z

[B20] Delage E. Puyaubert J. Zachowski A. Ruelland E. (2013). Signal transduction pathways involving phosphatidylinositol 4-phosphate and phosphatidylinositol 4,5-bisphosphate: convergences and divergences among eukaryotic kingdoms. *Prog. Lipid Res.* 52 1–14. 10.1016/j.plipres.2012.08.003 22981911

[B21] Devaiah S. P. Roth M. R. Baughman E. Li M. Tamura P. Jeannotte R. (2006). Quantitative profiling of polar glycerolipid species from organs of wild-type arabidopsis and a *PHOSPHOLIPASE Dα1* knockout mutant. *Phytochemistry* 67 1907–1924. 10.1016/j.phytochem.2006.06.005 16843506

[B22] Dörmann P. Benning C. (2002). Galactolipids rule in seed plants. *Trends Plant Sci.* 7 112–118. 11906834 10.1016/s1360-1385(01)02216-6

[B23] Douce R. (1974). Site of biosynthesis of galactolipids in spinach chloroplasts. *Science* 183 852–853. 10.1126/science.183.4127.852 17780772

[B24] Elkahoui S. Smaoui A. Zarrouk M. Ghrir R. Limam F. (2004). Salt-induced lipid changes in *Catharanthus roseus* cultured cell suspensions. *Phytochemistry* 65 1911–1917. 10.1016/j.phytochem.2004.06.021 15279997

[B25] Exton J. H. (1990). Signaling through phosphatidylcholine breakdown. *J. Biol. Chem.* 265 1–4. 2104616

[B26] Fujita M. Fujita Y. Noutoshi Y. Takahashi F. Narusaka Y. Yamaguchi-Shinozaki K. (2006). Crosstalk between abiotic and biotic stress responses: a current view from the points of convergence in the stress signaling networks. *Curr. Opin. Plant Biol.* 9 436–442. 10.1016/j.pbi.2006.05.014 16759898

[B27] Gigon A. Matos A.-R. Laffray D. Zuily-Fodil Y. Pham-Thi A.-T. (2004). Effect of drought stress on lipid metabolism in the leaves of *Arabidopsis thaliana* (Ecotype Columbia). *Ann. Bot.* 94 345–351. 10.1093/aob/mch150 15277243 PMC4242175

[B28] Gogna M. Bhatla S. C. (2020). Salt-tolerant and -sensitive seedlings exhibit noteworthy differences in lipolytic events in response to salt stress. *Plant Signal. Behav.* 15:1737451. 10.1080/15592324.2020.1737451 32141358 PMC7194373

[B29] Guo Q. Liu L. Barkla B. J. (2019). Membrane lipid remodeling in response to salinity. *Int. J. Mol. Sci.* 20:4264. 10.3390/ijms20174264 31480391 PMC6747501

[B30] Guschina I. A. Everard J. D. Kinney A. J. Quant P. A. Harwood J. L. (2014). Studies on the regulation of lipid biosynthesis in plants: application of control analysis to soybean. *Biochim. Biophys. Acta Biomembr.* 1838 1488–1500. 10.1016/j.bbamem.2014.02.008 24565795

[B31] Hasanuzzaman M. Nahar K. Rahman A. Mahmud J. A. Hossain M. S. Fujita M. (2016). *“Soybean production and environmental stresses,” in Environmental stresses in soybean production.* Amsterdam, Netherlands: Elsevier, 61–102. 10.1016/B978-0-12-801535-3.00004-8

[B32] He M. Qin C.-X. Wang X. Ding N.-Z. (2020). Plant unsaturated fatty acids: biosynthesis and regulation. *Front. Plant Sci.* 11:390. 10.3389/fpls.2020.00390 PMC7212373 32425958

[B33] Heilmann M. Heilmann I. (2015). Plant phosphoinositides complex networks controlling growth and adaptation. *Biochim. Biophys. Acta* 1851 759–769. 10.1016/j.bbalip.2014.09.018 25280638

[B34] Higashi Y. Okazaki Y. Myouga F. Shinozaki K. Saito K. (2015). Landscape of the lipidome and transcriptome under heat stress in *Arabidopsis thaliana*. *Sci. Rep.* 5:10533. 10.1038/srep10533 26013835 PMC4444972

[B35] Hoagland D. R. Arnon D. I. (1950). The water-culture method for growing plants without soil. *Circular. California Agric. Exp. Station* 347:32.

[B36] Hölzl G. Dörmann P. (2019). Chloroplast lipids and their biosynthesis. *Annu. Rev. Plant Biol.* 70 51–81. 10.1146/annurev-arplant-050718-100202 30786236

[B37] Hossain Z. Khatoon A. Komatsu S. (2013). Soybean proteomics for unraveling abiotic stress response mechanism. *J. Proteome Res.* 12 4670–4684. 10.1021/pr400604b 24016329

[B38] Ishiguro S. Kawai-Oda A. Ueda J. Nishida I. Okada K. (2001). The *DEFECTIVE IN ANTHER DEHISCENCE1* gene encodes a novel phospholipase A1 catalyzing the initial step of jasmonic acid biosynthesis, which synchronizes pollen maturation, anther dehiscence, and flower opening in Arabidopsis. *Plant Cell.* 13 2191–2209. 10.1105/tpc.13.10.2191 11595796 PMC139153

[B39] Ji W. Cong R. Li S. Li R. Qin Z. Li Y. (2016). Comparative proteomic analysis of soybean leaves and roots by iTRAQ provides insights into response mechanisms to short-term salt stress. *Front. Plant Sci.* 7:573. 10.3389/fpls.2016.00573 27200046 PMC4850148

[B40] Käll L. Canterbury J. D. Weston J. Noble W. S. MacCoss M. J. (2007). Semi-supervised learning for peptide identification from shotgun proteomics datasets. *Nat. Methods* 4 923–925. 10.1038/nmeth1113 17952086

[B41] Karki N. Johnson B. S. Bates P. D. (2019). Metabolically distinct pools of phosphatidylcholine are involved in trafficking of fatty acids out of and into the chloroplast for membrane production. *Plant Cell.* 31 2768–2788. 10.1105/tpc.19.00121 31511316 PMC6881139

[B42] Katam R. Shokri S. Murthy N. Singh S. K. Suravajhala P. Khan M. N. (2020). Proteomics, physiological, and biochemical analysis of cross tolerance mechanisms in response to heat and water stresses in soybean. *PLoS One* 15:e0233905. 10.1371/journal.pone.0233905 32502194 PMC7274410

[B43] Kosová K. Prášil I. Vítámvás P. (2013). Protein contribution to plant salinity response and tolerance acquisition. *Int. J. Mol. Sci.* 14 6757–6789. 10.3390/ijms14046757 23531537 PMC3645664

[B44] Lam H.-M. Xu X. Liu X. Chen W. Yang G. Wong F.-L. (2010). Resequencing of 31 wild and cultivated soybean genomes identifies patterns of genetic diversity and selection. *Nat. Genet.* 42 1053–1059. 10.1038/ng.715 21076406

[B45] Li M. Guo R. Jiao Y. Jin X. Zhang H. Shi L. (2017). Comparison of salt tolerance in soja based on metabolomics of seedling roots. *Front. Plant Sci.* 8:1101. 10.3389/fpls.2017.01101 28690628 PMC5481370

[B46] Liu A. Xiao Z. Li M.-W. Wong F.-L. Yung W.-S. Ku Y.-S. (2019). Transcriptomic reprogramming in soybean seedlings under salt stress. *Plant. Cell. Environ.* 42 98–114. 10.1111/pce.13186 29508916

[B47] Liu X. Zhai S. Zhao Y. Sun B. Liu C. Yang A. (2013). Overexpression of the phosphatidylinositol synthase gene (*ZmPIS*) conferring drought stress tolerance by altering membrane lipid composition and increasing ABA synthesis in maize. *Plant Cell. Environ.* 36 1037–1055. 10.1111/pce.12040 23152961

[B48] Loewus F. A. Murthy P. P. N. (2000). *myo*-Inositol metabolism in plants. *Plant Sci.* 150 1–19. 10.1016/S0168-9452(99)00150-8

[B49] Lv D.-W. Subburaj S. Cao M. Yan X. Li X. Appels R. (2013). Proteome and phosphoproteome characterization reveals new response and defense mechanisms of *Brachypodium distachyon* leaves under salt stress. *Mol. Cell. Proteomics* 13 632–652. 10.1074/mcp.m113.030171 24335353 PMC3916659

[B50] Magdy M. Mansour F. Hasselt P. R. Kuiper P. J. C. (1994). Plasma membrane lipid alterations induced by NaCl in winter wheat roots. *Physiol. Plant.* 92 473–478. 10.1111/j.1399-3054.1994.tb08838.x

[B51] Martin T. F. J. (1998). Phosphoinositide lipids as signaling molecules: common themes for signal transduction, cytoskeletal regulation, and membrane trafficking. *Annu. Rev. Cell. Dev. Biol.* 14 231–264. 10.1146/annurev.cellbio.14.1.231 9891784

[B52] McLoughlin F. Augustine R. C. Marshall R. S. Li F. Kirkpatrick L. D. Otegui M. S. (2018). Maize multi-omics reveal roles for autophagic recycling in proteome remodelling and lipid turnover. *Nat. Plants* 4 1056–1070. 10.1038/s41477-018-0299-2 30478358

[B53] Mikami K. Katagiri T. Luchi S. Yamaguchi-Shinozaki K. Shinozaki K. (1998). A gene encoding phosphatidylinositol-4-phosphate 5-kinase is induced by water stress and abscisic acid in *Arabidopsis thaliana*. *Plant J.* 15 563–568. 10.1046/j.1365-313X.1998.00227.x 9753781

[B54] Mizusawa N. Wada H. (2012). The role of lipids in photosystem II. *Biochim. Biophys. Acta Bioenerg.* 1817 194–208. 10.1016/j.bbabio.2011.04.008 21569758

[B55] Munns R. Tester M. (2008). Mechanisms of salinity tolerance. *Annu. Rev. Plant Biol.* 59 651–681. 10.1146/annurev.arplant.59.032607.092911 18444910

[B56] Nelson D. E. Rammesmayer G. Bohnert H. J. (1998). Regulation of cell-specific inositol metabolism and transport in plant salinity tolerance. *Plant Cell.* 10 753–764. 10.2307/3870662 9596634 PMC144026

[B57] Norberg P. Liljenberg C. (1991). Lipids of plasma membranes prepared from oat root cells. *Plant Physiol.* 96 1136–1141. 10.1104/pp.96.4.1136 16668310 PMC1080905

[B58] Nouairi I. Ammar W. B. Youssef N. B. Daoud D. B. Ghorbal M. H. Zarrouk M. (2006). Comparative study of cadmium effects on membrane lipid composition of *Brassica juncea* and *Brassica napus* leaves. *Plant Sci.* 170 511–519. 10.1016/j.plantsci.2005.10.003

[B59] Oliveros J. C. (2007). *VENNY. An interactive tool for comparing lists with Venn Diagrams.* Available Online at: http://bioinfogp.cnb.csic.es/tools/venny/index.html.bioinfogp.cnb.csic.es/tools/venny/index.html

[B60] Pang Q. Chen S. Dai S. Chen Y. Wang Y. Yan X. (2010). Comparative proteomics of salt tolerance in *Arabidopsis thaliana* and *Thellungiella halophila*. *J. Proteome Res.* 9 2584–2599. 10.1021/pr100034f 20377188

[B61] Pappan K. Austin-Brown S. Chapman K. D. Wang X. (1998). Substrate selectivities and lipid modulation of plant phospholipase Dα, -β, and -γ. *Arch. Biochem. Biophys.* 353 131–140. 10.1006/abbi.1998.0640 9578608

[B62] Parvaiz A. Satyawati S. (2008). Salt stress and phyto-biochemical responses of plants – a review. *Plant Soil Environ.* 54 89–99. 10.17221/2774-PSE

[B63] Phang T.-H. Shao G. Lam H.-M. (2008). Salt tolerance in soybean. *J. Integr. Plant Biol.* 50 1196–1212. 10.1111/j.1744-7909.2008.00760.x 19017107

[B64] Pical C. Westergren T. Dove S. K. Larsson C. Sommarin M. (1999). Salinity and hyperosmotic stress induce rapid increases in phosphatidylinositol 4, 5-bisphosphate, diacylglycerol pyrophosphate, and phosphatidylcholine in *Arabidopsis thaliana* cells. *J. Biol. Chem.* 274 38232–38240. 10.1074/jbc.274.53.38232 10608898

[B65] Qi X. Li M.-W. Xie M. Liu X. Ni M. Shao G. (2014). Identification of a novel salt tolerance gene in wild soybean by whole-genome sequencing. *Nat. Commun.* 5:4340. 10.1038/ncomms5340 25004933 PMC4104456

[B66] Rottet S. Besagni C. Kessler F. (2015). The role of plastoglobules in thylakoid lipid remodeling during plant development. *Biochim. Biophys. Acta Bioenerg.* 1847 889–899. 10.1016/j.bbabio.2015.02.002 25667966

[B67] Schertl P. Braun H.-P. (2014). Respiratory electron transfer pathways in plant mitochondria. *Front. Plant Sci.* 5:163. 10.3389/fpls.2014.00163 24808901 PMC4010797

[B68] Shelden M. C. Dias D. A. Jayasinghe N. S. Bacic A. Roessner U. (2016). Root spatial metabolite profiling of two genotypes of barley (Hordeum vulgare L.) reveals differences in response to short-term salt stress. *J. Exp. Bot.* 67 3731–3745. 10.1093/jxb/erw059 26946124 PMC4896359

[B69] Shiva S. Enninful R. Roth M. R. Tamura P. Jagadish K. Welti R. (2018). An efficient modified method for plant leaf lipid extraction results in improved recovery of phosphatidic acid. *Plant Methods* 14:14. 10.1186/s13007-018-0282-y 29449874 PMC5812192

[B70] Shoemaker S. D. Vanderlick T. K. (2002). Stress-induced leakage from phospholipid vesicles: effect of membrane composition. *Ind. Eng. Chem. Res.* 41 324–329. 10.1021/ie010049t

[B71] Shu S. Guo S.-R. Sun J. Yuan L.-Y. (2012). Effects of salt stress on the structure and function of the photosynthetic apparatus in *Cucumis sativus* and its protection by exogenous putrescine. *Physiol. Plant.* 146 285–296. 10.1111/j.1399-3054.2012.01623.x 22452600

[B72] Simidjiev I. Stoylova S. Amenitsch H. Javorfi T. Mustardy L. Laggner P. (2000). Self-assembly of large, ordered lamellae from non-bilayer lipids and integral membrane proteins in vitro. *Proc. Natl. Acad. Sci.* 97 1473–1476. 10.1073/pnas.97.4.1473 10677486 PMC26458

[B73] Smyth G. K. Speed T. (2003). Normalization of cDNA microarray data. *Methods*. 31 265–273. 10.1016/S1046-2023(03)00155-5 14597310

[B74] Stenzel I. Ischebeck T. König S. Hołubowska A. Sporysz M. Hause B. (2008). The type B phosphatidylinositol-4-phosphate 5-kinase 3 is essential for root hair formation in *Arabidopsis thaliana*. *Plant Cell* 20, 124–141. 10.1105/tpc.107.052852 18178770 PMC2254927

[B75] Sui N. Han G. (2014). Salt-induced photoinhibition of PSII is alleviated in halophyte *Thellungiella halophila* by increases of unsaturated fatty acids in membrane lipids. *Acta Physiol. Plant.* 36 983–992. 10.1007/s11738-013-1477-5

[B76] Tasseva G. Richard L. Zachowski A. (2004). Regulation of phosphatidylcholine biosynthesis under salt stress involves choline kinases in *Arabidopsis thaliana*. *FEBS Lett.* 566 115–120. 10.1016/j.febslet.2004.04.015 15147879

[B77] Tian T. Liu Y. Yan H. You Q. Yi X. Du Z. (2017). AgriGO v2.0: A GO analysis toolkit for the agricultural community, 2017 update. *Nucleic Acids Res.* 45 W122–W129. 10.1093/nar/gkx382 28472432 PMC5793732

[B78] Torres-Franklin M.-L. Gigon A. de Melo D. F. Zuily-Fodil Y. Pham-Thi A.-T. (2007). Drought stress and rehydration affect the balance between MGDG and DGDG synthesis in cowpea leaves. *Physiol. Plant* 131 201–210. 10.1111/j.1399-3054.2007.00943.x 18251892

[B79] Torres-Romero D. Gomez-Zambrano A. Serrato A. J. Sahrawy M. Merida A. (2020). Fibrillin 2 interacts with other proteins to protect photosystem II against abiotic stress in *Arabidopsis thaliana*. *bioRxiv.* 10.1101/2020.10.07.329979

[B80] Tsydendambaev V. D. Ivanova T. V. Khalilova L. A. Kurkova E. B. Myasoedov N. A. Balnokin Y. V. (2013). Fatty acid composition of lipids in vegetative organs of the halophyte *Suaeda altissima* under different levels of salinity. *Russ. J. Plant Physiol.* 60 661–671. 10.1134/S1021443713050142

[B81] Tuteja N. Sopory S. K. (2008). Chemical signaling under abiotic stress environment in plants. *Plant Signal. Behav.* 3 525–536. 10.4161/psb.3.8.6186 19513246 PMC2634487

[B82] Wang H. Zhang M. Guo R. Shi D. Liu B. Lin X. (2012). Effects of salt stress on ion balance and nitrogen metabolism of old and young leaves in rice (*Oryza sativa* L.). *BMC Plant Biol.* 12:194. 10.1186/1471-2229-12-194 23082824 PMC3496643

[B83] Wang K. Guo Q. Froehlich J. E. Hersh H. L. Zienkiewicz A. Howe G. A. (2018). Two abscisic acid-responsive plastid lipase genes involved in jasmonic acid biosynthesis in *Arabidopsis thaliana*. *Plant Cell.* 30 1006–1022. 10.1105/tpc.18.00250 29666162 PMC6002186

[B84] Wang T. Tohge T. Ivakov A. Mueller-Roeber B. Fernie A. R. Mutwil M. (2015). Salt-related *MYB1* coordinates abscisic acid biosynthesis and signaling during salt stress in arabidopsis. *Plant Physiol.* 169 1027–1041. 10.1104/pp.15.00962 26243618 PMC4587467

[B85] Wang Y. Zhang X. Huang G. Feng F. Liu X. Guo R. (2020). Dynamic changes in membrane lipid composition of leaves of winter wheat seedlings in response to PEG-induced water stress. *BMC Plant Biol.* 20:84. 10.1186/s12870-020-2257-1 32085729 PMC7035713

[B86] Welti R. Li W. Li M. Sang Y. Biesiada H. Zhou H.-E. (2002). Profiling membrane lipids in plant stress responses. *J. Biol. Chem.* 277 31994–32002. 10.1074/jbc.M205375200 12077151

[B87] Xie C. Mao X. Huang J. Ding Y. Wu J. Dong S. (2011). KOBAS 2.0: a web server for annotation and identification of enriched pathways and diseases. *Nucleic Acids Res.* 39 W316–W322. 10.1093/nar/gkr483 21715386 PMC3125809

[B88] Xiong J. Sun Y. Yang Q. Tian H. Zhang H. Liu Y. (2017). Proteomic analysis of early salt stress responsive proteins in alfalfa roots and shoots. *Proteome Sci.* 15 1–19. 10.1186/s12953-017-0127-z 29093645 PMC5663070

[B89] Yamane K. Rahman S. Kawasaki M. Taniguchi M. Miyake H. (2004). Pretreatment with a low concentration of methyl viologen decreases the effects of salt stress on chloroplast ultrastructure in rice leaves (*Oryza sativa* L.). *Plant Prod. Sci.* 7 435–441. 10.1626/pps.7.435

[B90] Yang J. I. Perera I. Y. Brglez I. Davis A. J. Stevenson-Paulik J. Phillippy B. Q. (2007). Increasing plasma membrane phosphatidylinositol(4,5)bisphosphate biosynthesis increases phosphoinositide metabolism in *Nicotiana tabacum*. *Plant Cell.* 19 1603–1616. 10.1105/tpc.107.051367 17496116 PMC1913725

[B91] Yekutieli D. Benjamini Y. (2001). The control of the false discovery rate in multiple testing under dependency. *Ann. Stat.* 29 1165–1188. 10.1214/aos/1013699998

[B92] Yin X. Komatsu S. (2017). Comprehensive analysis of response and tolerant mechanisms in early-stage soybean at initial-flooding stress. *J. Proteomics.* 169 225–232. 10.1016/j.jprot.2017.01.014 28137666

[B93] Yoeun S. Cho K. Han O. (2018). Structural evidence for the substrate channeling of rice allene oxide cyclase in biologically analogous nazarov reaction. *Front. Chem.* 6:500. 10.3389/fchem.2018.00500 30425978 PMC6218421

[B94] Youssef A. Laizet Y. Block M. A. Maréchal E. Alcaraz J. P. Larson T. R. (2010). Plant lipid-associated fibrillin proteins condition jasmonate production under photosynthetic stress. *Plant J.* 61 436–445. 10.1111/j.1365-313X.2009.04067.x 19906042

[B95] Zhang B. Kuster B. (2019). Proteomics is not an island: multi-omics integration is the key to understanding biological systems. *Mol. Cell. Proteomics* 18 S1–S4. 10.1074/mcp.E119.001693 31399542 PMC6692779

[B96] Zhang J. Yang D. Li M. Shi L. (2016). Metabolic profiles reveal changes in wild and cultivated soybean seedling leaves under salt stress. *PLoS One* 11:e0159622. 10.1371/journal.pone.0159622 27442489 PMC4956222

[B97] Zhang Z. Mao C. Shi Z. Kou X. (2017). The amino acid metabolic and carbohydrate metabolic pathway play important roles during salt-stress response in tomato. *Front. Plant Sci.* 8:1231. 10.3389/fpls.2017.01231 28769946 PMC5511834

[B98] Zhao Y. Dong W. Zhang N. Ai X. Wang M. Huang Z. (2014). A wheat allene oxide cyclase gene enhances salinity tolerance via jasmonate signaling. *Plant Physiol.* 164, 1068–1076. 10.1104/pp.113.227595 24326670 PMC3912080

