# Supplementary material for: Galactolipid and Phospholipid Profile and Proteome Alterations in Soybean Leaves at the Onset of Salt Stress
Source: Front Plant Sci. 2021 Mar 17;12:644408. doi: 10.3389/fpls.2021.644408 (PMC8010258; doi:10.3389/fpls.2021.644408)
Supplement: Supplementary file 1 [file Data_Sheet_1.DOCX]

Supplementary Material

**Supplementary Figure S1** Composition of major lipid species in soybean leaf during salt stress

**
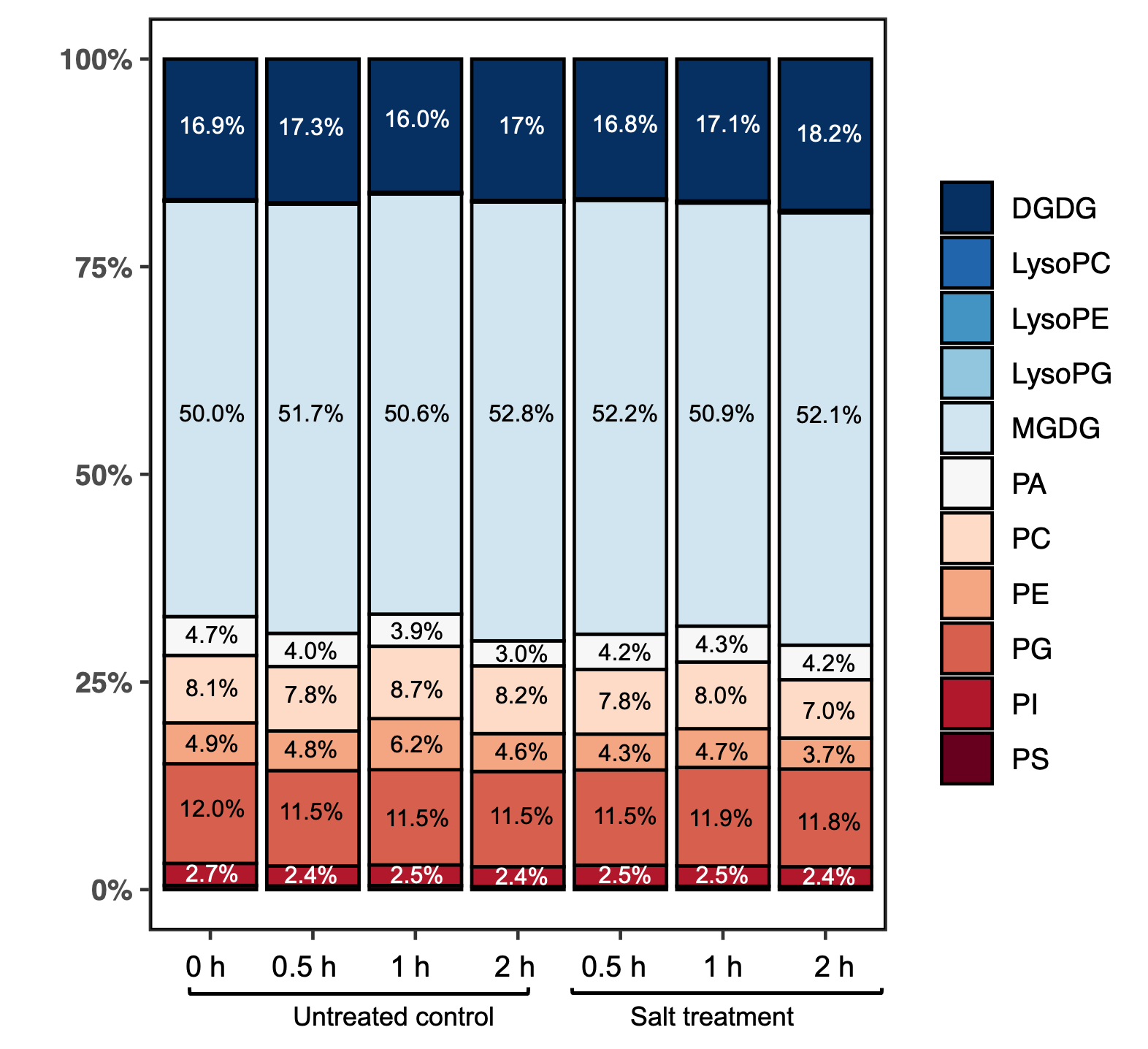
**

**Supplementary Figure S2** Changes in the ratio of DGDG/MGDG with or without salt treatment

*, significant difference at *p* ≤ 0.05; **, significant difference at *p* ≤ 0.01 using the Student’s t-test.

**Supplementary Figure S3** The volcano plots of differentially expressed proteins assigned by subcellular localisation


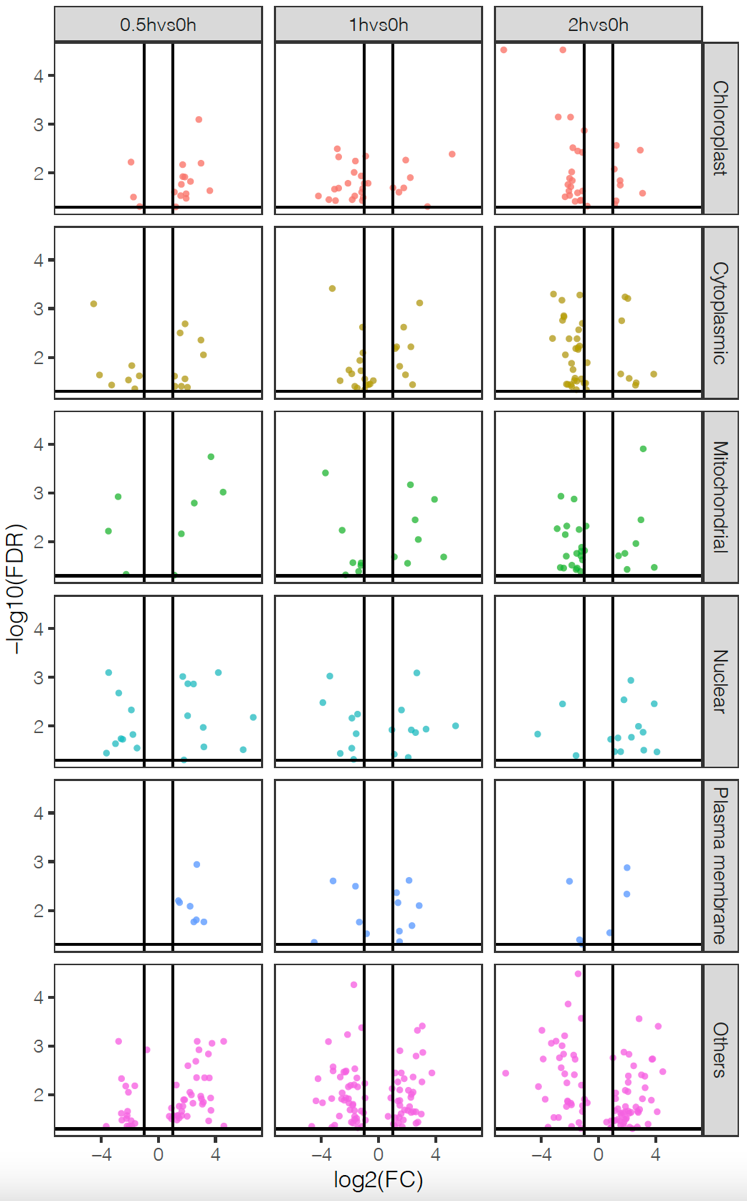


Log_2_-fold changes (stress vs normal control) of each protein is plotted against its -log_10_ adjusted *p*-value ≤ 0.05. The horizontal line indicates adjusted *p*-value = 0.05. The vertical line indicates fold change ≥ 1.

**Supplementary Table S1** Raw data of lipid profiling analysis (submitted separately).

**Supplementary Table S2** Changes in ratio of PC/PE with different lipid acyl species under salt stress

| **Fold change of ratio (PC/PE)**  **(NaCl vs CK)** | **0.5h** | **1h** | **2h** |
| --- | --- | --- | --- |
| PC/PE (34:3) | 1.1276 | **1.2234*** | 1.066 |
| PC/PE (34:2) | 1.1018 | **1.2704**** | 1.0557 |
| PC/PE (34:1) | 1.4259 | 0.9257 | 0.8586 |
| PC/PE (36:6) | 1.2988 | **1.4094**** | 1.1507 |
| PC/PE (36:5) | 1.0858 | **1.1913**** | 1.0622 |
| PC/PE (36:4) | 1.1611 | 1.1275 | 1.069 |
| PC/PE (36:3) | 1.0191 | 1.1558 | 0.9946 |
| PC/PE (36:2) | 1.0678 | **1.2852**** | 1.0256 |

*, significant difference at *p* ≤ 0.05; **, significant difference at *p* ≤ 0.01 using the Student’s t-test.

**Supplementary Table S3** Changes in unsaturation index of various lipid classes in soybean during salt stress

| **Lipid class** |  | **Untreated control** | | | **Salt treatment** | | |
| --- | --- | --- | --- | --- | --- | --- | --- |
|  | **0h** | **0.5h** | **1h** | **2h** | **0.5h** | **1h** | **2h** |
| DGDG | 4.94 | 4.78 | 4.95 | 4.48 | 4.77 | 4.93 | 4.34 |
| MGDG | 5.82 | 5.83 | 5.83 | 5.83 | 5.82 | 5.81 | **5.82*** |
| PG | 1.58 | 1.62 | 1.65 | 2.15 | 1.65 | 1.62 | 2.17 |
| PE | 2.90 | 2.91 | 2.92 | 2.94 | 2.91 | 2.90 | **2.87**** |
| PC | 3.08 | 3.07 | 3.09 | 3.07 | 3.08 | **3.03*** | **2.99**** |
| PI | 2.50 | 2.52 | 2.52 | 2.54 | 2.51 | 2.51 | 2.51 |
| PS | 2.14 | 2.20 | 2.19 | 2.18 | 2.16 | **2.12*** | 2.20 |
| PA | 2.91 | 2.94 | 2.93 | 2.86 | 2.93 | 2.91 | **2.85*** |
| LysoPC | 1.63 | 1.81 | 1.61 | 1.63 | 1.69 | 1.62 | 1.66 |

*, significant difference at *p* ≤ 0.05; **, significant difference at *p* ≤ 0.01 using the Student’s t-test.

**Supplementary Table S4** Protein lists of each KEGG enrichment pathway (submitted separately).

**Supplementary Table S5** Differentially expressed proteins identified by Orbitrap LC-MS/MS at 0.5, 1 and 2 h of salt treatment (submitted separately).

**Supplementary Table S6** GO enrichment of differentially expressed proteins from 0.5, 1 and 2 h (submitted separately).

**Supplementary Table S7** Selective stress-related proteins and their corresponding expression after salt treatment


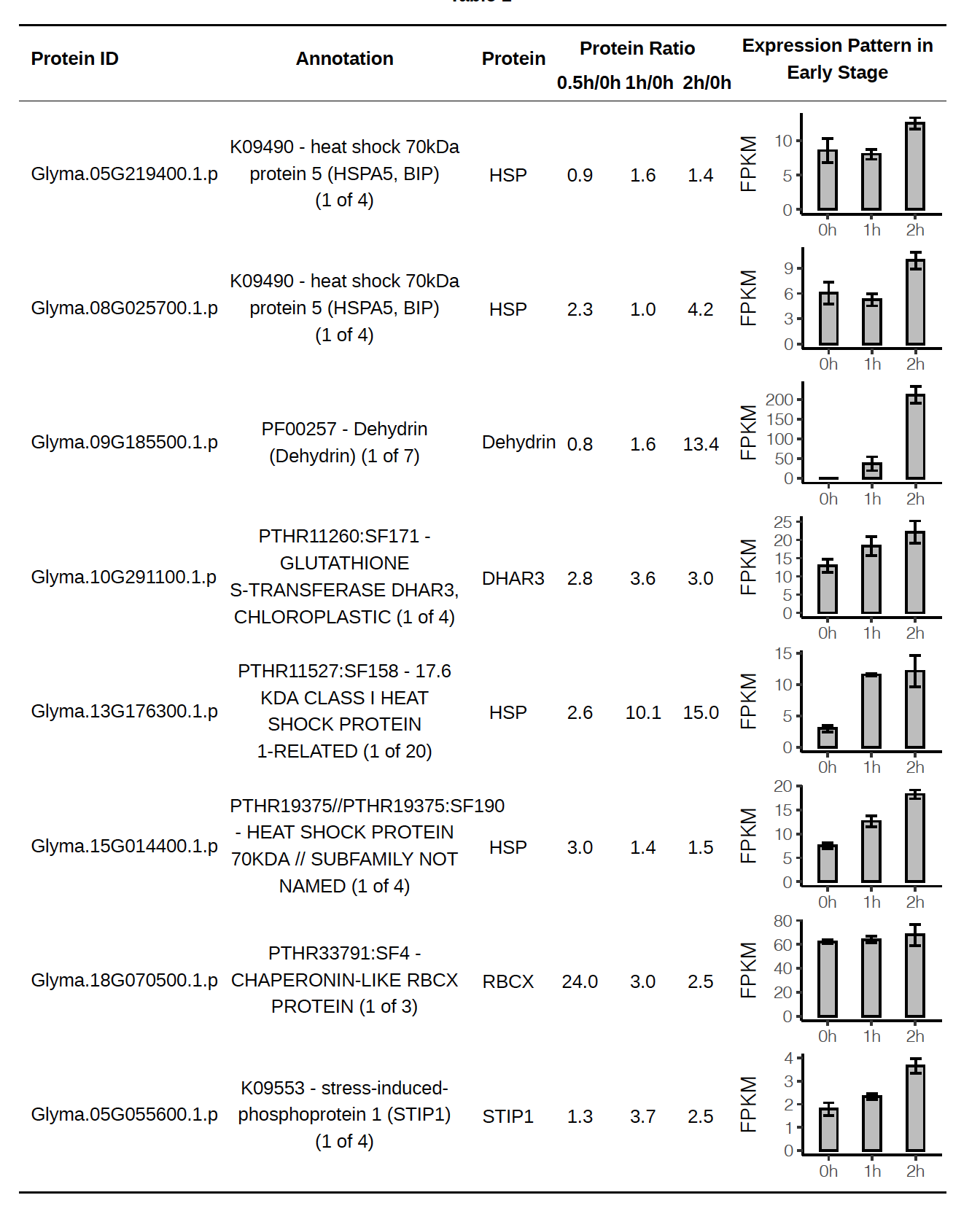


**Supplementary Table S8** Differentially expressed proteins shown in Figures. 4, 5 and 6 (submitted separately).

**Supplementary Table S9** Label free quantification proteome raw data of all time points in C08 leaves (submitted separately).
